# An Investigation on the Aggregation and Rheodynamics of Human Red Blood Cells Using High Performance Computations

**DOI:** 10.1155/2017/6524156

**Published:** 2017-04-04

**Authors:** Dong Xu, Chunning Ji, Eldad Avital, Efstathios Kaliviotis, Ante Munjiza, John Williams

**Affiliations:** ^1^State Key Laboratory of Hydraulic Engineering Simulation and Safety, Tianjin University, Weijin Road, Tianjin 300072, China; ^2^School of Engineering & Materials Science, Queen Mary University of London, Mile End Road, London E1 4NS, UK; ^3^Department of Mechanical Engineering and Materials Science and Engineering, Faculty of Engineering and Technology, Cyprus University of Technology, 45 Kitiou Kyprianou, 3041 Limassol, Cyprus; ^4^Faculty of Civil Engineering, University of Split, Split, Croatia

## Abstract

Studies on the haemodynamics of human circulation are clinically and scientifically important. In order to investigate the effect of deformation and aggregation of red blood cells (RBCs) in blood flow, a computational technique has been developed by coupling the interaction between the fluid and the deformable RBCs. Parallelization was carried out for the coupled code and a high speedup was achieved based on a spatial decomposition. In order to verify the code's capability of simulating RBC deformation and transport, simulations were carried out for a spherical capsule in a microchannel and multiple RBC transport in a Poiseuille flow. RBC transport in a confined tube was also carried out to simulate the peristaltic effects of microvessels. Relatively large-scale simulations were carried out of the motion of 49,512 RBCs in shear flows, which yielded a hematocrit of 45%. The large-scale feature of the simulation has enabled a macroscale verification and investigation of the overall characteristics of RBC aggregations to be carried out. The results are in excellent agreement with experimental studies and, more specifically, both the experimental and simulation results show uniform RBC distributions under high shear rates (60–100/s) whereas large aggregations were observed under a lower shear rate of 10/s.

## 1. Introduction

The red blood cell (RBC, also referred to as erythrocyte) is the most common type of cell occurring in human blood and occupies approximately 45% of the total blood volume for man and 40% for women. RBCs in a healthy state have a biconcave shape with a diameter of 6–8 *μ*m and a thickness of about 2 *μ*m at the edges and about 1 *μ*m at the center [1]. The RBC aggregation, which is the mechanism that greatly influences the non-Newtonian properties of blood [2], occurs when the shear forces are low and cells attract each other to form rouleaux (structures resembling coin piles), larger aggregates, and networks of aggregates. RBC aggregation can cause complications in health related issues; therefore, the investigation of deformability and aggregation of RBCs in blood flow can be promising for the better understanding and diagnosis of many diseases in clinical medicine.

Due to the complex mechanism of fluid-structure interaction, the theoretical analysis of RBCs or capsules is difficult and is usually limited to simple geometries and small deformations (Barthes-Biesel, 1980) and an alternative approach—numerical simulation—has attracted much attention. To date, most simulations have tended to target a relatively small number of cells ([3]; Dupin et al., 2006), due to their complex nature. The intrinsic complexity of biological systems requires a closer combination between experimental and computational approaches ([3, 4], Secomb, 2011). This requires large-scale simulations because experiments usually measure the macroscale effects with large quantities of cells [4–7].

So far, most RBC simulations have tended to target a relatively small number of cells [3, 8, 9]. However, it is often said that biological systems, such as cells, are “complex systems.” Such complex systems involve not only different categories of cells but also very large numbers of simple and identical elements interacting to produce “complex” behaviour. Experimental results show that the number of RBCs involved in the formation of aggregates and aggregate network structures in blood flow is much greater compared to those used in the aforementioned studies [7]. Although advances in accurate, quantitative experimental approaches will doubtlessly continue, insights into the functioning of biological systems will not result from purely intuitive assaults. This is because of the intrinsic complexity of biological systems and a combination of experimental and computational approaches is expected to resolve this problem [3, 4]. This again requires large-scale simulations because experiments usually measure the macroscale effects with large quantities of cells involved [4–7]. Large sale simulations of RBCs developed rapidly in recent years [10–12]. With the support of modern GPUs, Tang and Karniadakis [13] conducted a large-scale simulation of spontaneous vesicle formation consisting of 128 million particles.

In this paper, we present our computational research on RBC aggregations [14]. A 3D computational modelling has been developed for the deformation and aggregation of the RBCs. With the support of high performance computers, the transport and aggregation of up to 49,152 RBCs, at a hematocrit of 45% in a simple shear flow, have been simulated. The large-scale nature of our computation also enabled us to perform a direct comparison of the macroscale aggregation feature observed in experiments with excellent agreement. Simulations were also carried out in a Poiseuille flow formed in a tube with a confined throat to investigate RBC transport in stenosed vessels or micro peristaltic pumps.

In this paper, computational research on RBC aggregations is presented in which large-scale computations have enabled direct comparisons of macroscale aggregation features with experimental observations.

## 2. Methodology

### 2.1. Solvers for the Fluid, the Solids, and Their Interactions

#### 2.1.1. A Solver for Incompressible Viscous Flow

In order to simulate incompressible viscous flow, we used the in-house Computational Fluid Dynamics (CFD) code, CgLes [16]. CgLes is a three-dimensional fluid solver with second-order accuracy in both time and space and is based on a finite volume formulation. The projection method was used to decouple flow velocities and pressure. The capability of CgLes to simulate both laminar and turbulent flow has been extensively verified [17–19]. An implicit Crank-Nicolson scheme for the time-stepping was used for the diffusion term. The advection term, though very small and often neglected, was retained and discretized with the explicit two-step Adams-Bashforth scheme.

#### 2.1.2. Modelling the Deformation of the RBCs

The combined finite-discrete element method (FEM-DEM) [20, 21] was used to simulate the movement and deformation of the RBCs under the various forces developed in the fluid. This method incorporates movements, deformations, contacts, and interactions of RBCs. More details on this method were presented by Munjiza [20]. The initial RBC shape was described by a biconcave function [22]:

(1) $$\begin{array}{c} y=0.5{\left(\;1-x^{2}\;\right)}^{1/2}\left(\;a_{0}+a_{1}x^{2}+a_{2}x^{4}\;\right),\;-1\leq x\leq 1, \end{array}$$

where *a*_0_ = 0.207, *a*_1_ = 2.002, and *a*_2_ = −1.122.

In the present study, the RBC membrane was treated as a thin solid shell, meshed with tetrahedron elements and treated as a hyperelastic material. The most widely used constitutive laws include the neo-Hookean model [23] and the Mooney–Rivlin model [24, 25], which was adopted following Y. L. Liu and W. K. Liu [24]. The deformation of the solid can be written as

(2) $$\begin{array}{c} x=f\left(\;p\;\right)=p+u\left(\;p\;\right), \end{array}$$

where **p** are positions of the material points, **f** is a smooth function mapping the initial material points to the deformed points, and **u**(**p**) is the displacement mapping. The change in deformation in the vicinity of each material point can be described by the deformation gradient tensor **F**:

(3) $$\begin{array}{c} F\left(\;p\;\right)=\nabla f\left(\;p\;\right)=I+\nabla u\left(\;p\;\right), \end{array}$$

where **I** is the identity matrix. The left hand side Cauchy-Green deformation tensor can be written as

(4) $$\begin{array}{c} B=FF^{T}. \end{array}$$

#### 2.1.3. Coupling the Fluid and the Solid Simulation

The fluid motion and solid deformation were coupled using an Immersed Boundary (IB) method [26] which links the interface between the fluid and the solid, both of which have independent meshes. By introducing a force term into the momentum equations of the fluid, the Immersed Boundary points serve as nonslip boundaries to the fluid solver. The surface nodes of the solids were then treated as Immersed Boundary points for the fluid to form nonslip wall boundaries. A direct-forcing scheme, which has been well established and verified [17], was used for the implementation of the IB method. With the body force due to the Immersed Boundary condition incorporated, the time-discretized momentum equation of the fluid can be written as

(5) $$\begin{array}{c} \frac{u^{n+1}-u^{n}}{\Delta t}={rhs}^{n+i/2}+f^{n+i/2}, \end{array}$$

where **rhs**^*n* + *i*/2^ regroups the convective, pressure, and viscous terms at the intermediate time level between *t*^*n*^ and *t*^*n*+1^. The force term which yields the desired velocity **u**^(*d*)^ then becomes

(6) $$\begin{array}{c} f^{n+i/2}=\frac{u^{\left(d\right)}-u^{n}}{\Delta t}-{rhs}^{n+i/2}. \end{array}$$

#### 2.1.4. Modelling the Interaction between RBCs

The adhesive force, which causes aggregation, originates from molecular forces such as van der Waals attractions, on the cell surfaces [27]. Cell-cell communications and molecular coupling may also contribute to formation of adhesive force. An accurate quantitative modelling of the adhesive force between RBCs is very important for the simulation of the RBC aggregations. The strength of the adhesion between two cells can usually be described by the adhesion work, *σ*, which is the work required to separate two adhered cells. The JKR model [28] was used in the present study to compute the adhesion forces, where the relationship between the adhesive force *F* and the penetration depth *h* is described by

(7) F=Ka3R−6πσKa3,h=a2R−236πσaK,

where *R* is the radius of the tip, *a* is the contact area radius, *σ* is the work of adhesion, and *K* is the effective Young modulus.

### 2.2. Parallelization and Implementation

In order to simulate accurately the macroscale behaviours of red blood cells, such as the formation and development of aggregations structures, there should be a sufficient number of red blood cells involved in the simulation. Parallelization of the solver is necessary for simulation of a large quantity of RBCs. The fluid code—CgLes—was parallelized using MPI and spatial decomposition and a high scalability has been fulfilled [16]. For RBCs, the decomposition of the entire computational domain shares the same block division with the fluid. The advantage of this scheme is that the fluid block and the solid block for the same physical space are always installed on the same processor. Therefore, no remote data transfer between the fluid and the solid is required. Thus, no global data operation is required and all datasets are only running on a local processor. The computation is fully scalable in the spatial domain, which makes simulation with very large scales (running on hundreds of cores) possible.

**Figure 1:**
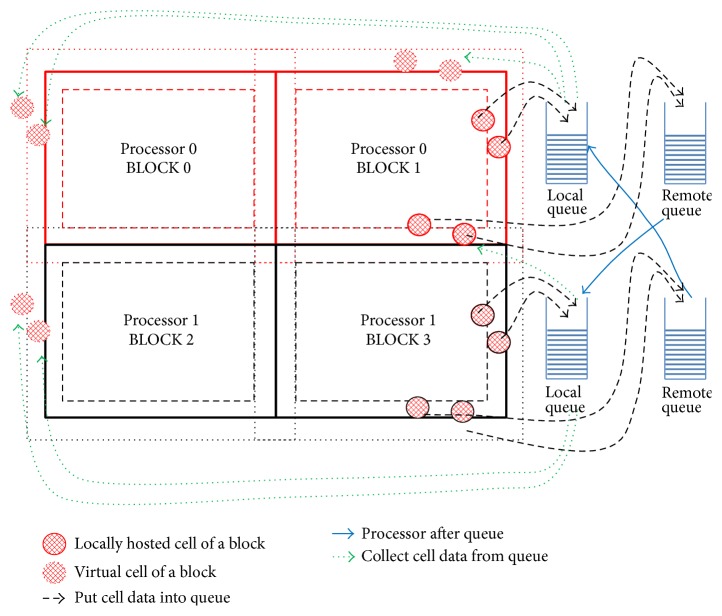
Schematic for parallelization of the solid solver for RBCs.

The procedure for the parallelization of the RBCs is as follows: (1) obtain the spatial decomposition by inheriting block and neighbourhood information from the fluid domain; (2) seed red blood cells randomly on each block and carry out initialization; (3) obtain the forces acting on boundary nodes of the solid, which are also the forces on Immersed Boundary points solved by the fluid code CgLes; (4) let each processor solve all cells hosted by its local blocks at time *t*; (5) find the cells located in the buffer areas of each block; (6) transfer the information of the cells in the buffer areas to a local queue or a remote queue depending on whether the neighbour block is local or remote; (7) exchange remote data information using MPI functions; (8) exchange local data information by collecting local queue information; (9) collect cell data posted by neighbours. If the collected cell is already found in local block, overwrite its data with collected data; if not, append a new cell; (10) delete cells outside of the buffer area; (11) go on with the next step by setting *t* = *t* + *dt* and repeat step (3). The scheme for parallelization of the solid solver for the RBCs is shown in Figure 1. For comparison, a scheme with nonbuffered data exchange (using MPI_BSEND) was also tested during implementation.

**Figure 2:**
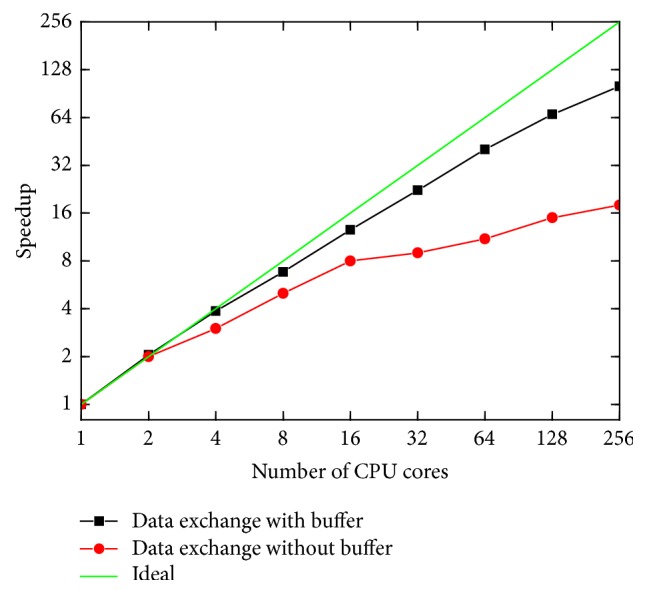
Speedup of the parallelized code with number of CPU cores.

The speedup of the parallelized code was tested using 512 RBCs in each computational block and the results are shown in Figure 2. It can be seen that, for the proposed buffered data exchange, the speedup of the code is quite close to the ideal case, namely, a linear speedup, and demonstrates good scalability. It also shows that the buffered data exchange yields much higher speedups at large-scale computations, especially when core numbers exceed 32.

As an example, we consider the simulation of RBC transport in shear flows (the case shown in Section 3.4) in which 512 RBCs were installed in each block at randomly allocated positions and with random initial velocities. The simulation ran on 96 cores of a Cray XE6 system. Each core has a 2.3 GHz 550 AMD Opteron processor with one computational block installed. Around 5 G memory was used on each core, and, for one time step, it took around 4 seconds of CPU time. For 50 seconds of physical time, the total CPU time is around 26 days. The interaction computation in the FEM-DEM solver consumes the most CUP time and the fluid solver took only 20% of the total time; see Table 1.

## 3. Results and Discussions

### 3.1. Simulation of a Capsule in a Poiseuille Flow

**Figure 3:**
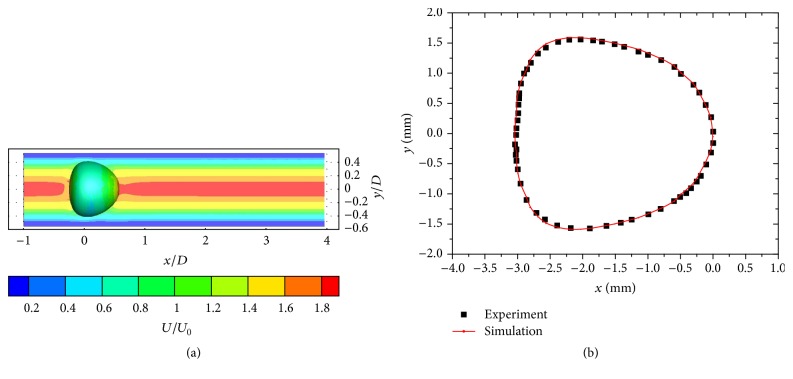
Simulation of a capsule passing through a microchannel: (a) contour of streamwise flow velocity (*U* is normalized with the mean velocity *U*_0_, and the coordinates are normalized with tube diameter *D*) and (b) comparison of the capsule deformation against experimental data [15].

In order to verify the code's capability in simulating the interaction between fluids and solids, a simulation was carried out for a capsule passing through a microchannel and the results were compared against the experiment by Risso et al. [15]. The test section consists of a horizontal glass tube with an inner radius *R* = 2 mm and a length of 220 mm. The suspending fluid is Rhodorsil 47V1000 Silicone oil, which is a Newtonian fluid with a density *ρ* = 970 kg/m^3^ and a viscosity *η* = 1.02 Pa·s. The numerical simulation was configured with parameters the same as those described in [15]. The capsule was artificially made and its constitutive property was fully tested and can be described as a hyperelastic membrane. The case C15 from [15] with capillary number of Ca = 0.015 was adopted in the simulation and the results are shown in Figure 3.

The flow is solved using fluid solver-CgLes and the tube was represented using Immersed Boundary points.  Figure 3(b) shows that the outline shape of the capsule from the simulation agrees very closely with that from the experiment and verifies the code's capability of simulating fluid-solid interactions, as well as the deformation of hyperelastic materials, which is crucial when RBCs experience significant deformations under high shear stress. Verification of the simulations of the adhesion between two RBCs was also carried out; see [12].

### 3.2. Simulation of RBC Motion in Poiseuille Flows

**Figure 4:**
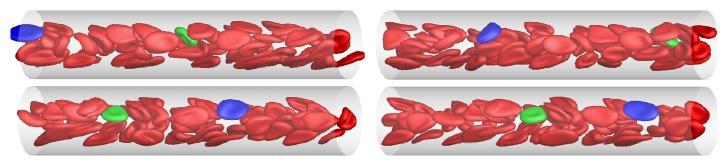
Snapshot of RBC motion (the hematocrit is 20%; blue: RBC near tube wall; green: RBC near tube center).

RBCs usually have complex patterns of motion in blood vessels due to the shearing of the background fluid and widely known motions include tank-trending, tumbling, and rotation. To verify the capability of our computational model in reproducing the correct RBC behaviour, a simulation was carried for a Poiseuille flow with hematocrit of 20% and typical computational results are shown in Figure 4. The tube diameter was 22.5 *μ*m and the mean flow velocity is 187.5 *μ*m/s. To make the motion more easily identifiable, two individual RBCs are shown in different colors.

**Figure 5:**
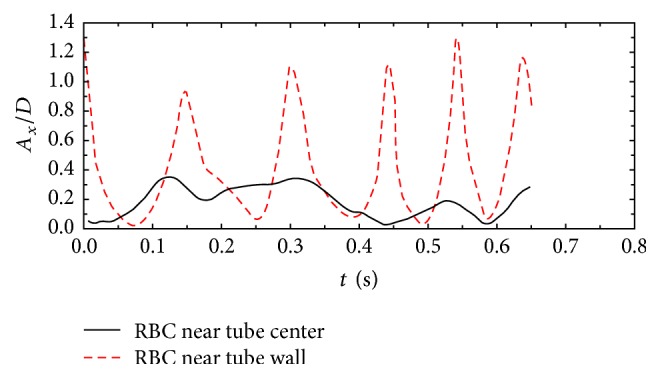
Time history of RBC orientation angle referring to the streamwise direction: *A*_*x*_ (*D* is the RBC diameter).

It can be seen that the RBCs near the tube wall tend to have a larger stretching ratio and a lower velocity than those near the center. The features of the RBC motions can also be identified by plotting the time history of orientation angle referring to tube axes; see Figure 5. It can be seen that the RBCs near the tube wall have more regular periodical motion patterns, which is a combination of tank-trending, tumbling, and rotation. This kind of motion is also widely reported by other researchers ([24]; Shi et al., 2012; Sui et al., 2008).

### 3.3. Simulation of the Deformation of RBCs through a Confined Tube

Human RBCs usually exhibit large deformations only when subjected to high fluid shear stresses and, under such circumstance, aggregation is difficult to form. Therefore, we simulated the deformation of RBCs in this part without considering cell-cell adhesion; namely, only repulsive forces were implemented when cell-cell contact occurs to prevent them from overlapping [20]. The simulation was carried out for a simple Poiseuille flow formed in a tube with a confined throat. The background for such cases includes diseased blood vessels (e.g., stenosed vessels) (Kamada, 2012) as well as micro peristaltic pumps (the coordinate is fixed on the rotor).

**Figure 6:**
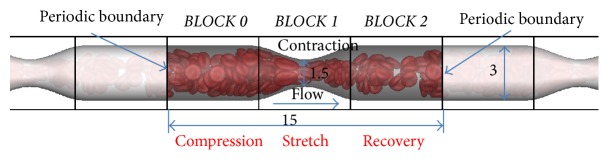
Schematic of the simulation on RBC transport through a confined tube (all sizes are normalized with the RBC diameter: *D*).

A structured fluid grid was adopted and the Immersed Boundary method was used to form the tube. The RBCs were treated as solid shells with hyperelastic material properties. The simulations were carried out in a 15*D* long tube with a diameter of 3*D* except in the middle, where the tube diameter shrinks to 1.5*D* at the throat. The fluid grid sizes were 0.1*D* in all three directions and the mesh size for the solid was 0.15*D* (*D* is the RBC diameter). Both the fluid and the RBC properties were the same as those in the above shear flow. The pressure gradient was 1000 dyn/cm^2^, and the hematocrit was 40%. The computational domain was divided into 3 blocks, namely, BLOCK 0, BLOCK 1, and BLOCK 2; see Figure 6. Periodic boundaries were adopted in the streamwise direction to simulate the wave train-like motion of micro peristaltic pumps.

**Figure 7:**
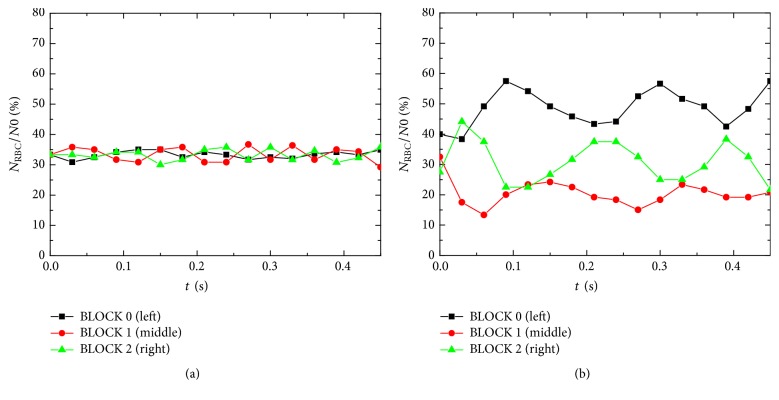
Comparison of RBC numbers in each computational block: (a) straight tube and (b) confined tube.

The simulations yielded abundant detailed information of RBC motions. In this paper, however, we focus on the streamwise distribution of RBCs. The number of RBCs in each computational block, *N*_RBC_, was counted continuously after sufficient mixing of the cells during the simulation. The interesting point is that although BLOCK 0 and BLOCK 2 are geometrically symmetrical (see Figure 6), the number of RBCs is quite different; see Figure 7(b). The block on the left of the throat (BLOCK 0) tends to hold 20% more RBCs on average compared with BLOCK 2. This implies that although the flow discharge is constant over every cross section of the tube, the velocity profile and RBC distribution are not symmetrical over the tube throat. The RBCs on the left of the throat are subjected to higher pressure from the fluid and are compressed. At the throat (BLOCK 1), the RBCs are subjected to higher shear stress and exhibit status of “stretching.” On the right of the throat (BLOCK 2), the RBCs recover. However, the statistics of RBC transport in a straight tube show quite uniform distribution of RBC along the tube; see Figure 7(a). Besides, Figure 7(b) also shows that the numbers of RBCs contained in each of the three blocks are not constant over time. This implies that the transport of RBCs through the tube throat is unsteady with some periodic pulses. One possible mechanism may be related to the dynamics of individual RBCs. The simulations show that, in stenosed vessels or micro peristaltic pumps, the hematocrit may not only vary in the longitudinal direction but also fluctuate with time.

The decrease in the average number of cells after the constriction may also illustrate the Fahraeus effect, that is, the decrease of the hematocrit with the decrease of vessel diameter in the microcirculation. Although in the present study the constriction does not lead to a decreased tube diameter the observed behaviour of the cells (stretching in the constriction) signifies the documented cell-structural role in the Fahraeus phenomenon.

### 3.4. Large-Scale Simulation of RBC Aggregation in a Shear Flow

**Figure 8:**
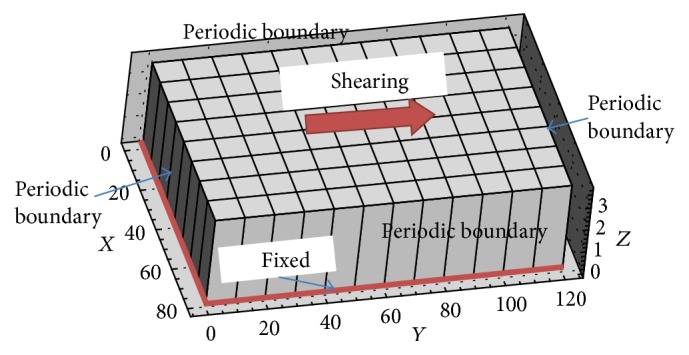
Computational domain and boundary conditions of the simulation (all coordinates were normalized with RBC diameter).

To investigate the large-scale structural characteristics of blood caused by the formation and development of RBC aggregations, simulations have been carried out with the same configuration as in the experiments performed by Kaliviotis and coworkers [5, 7, 14]. The experiment was a shearing system where the flow was created between two rotating glass plates separated by a gap of *h* = 30 *μ*m. The lower plate was driven by a stepper motor to create a shear plane in the direction vertical to the bottom plate with a nominal shear rate *γ*. The center of the viewing and measurement window was located at a radius *R* = 7500 *μ*m from the axis of rotation. Whole blood with a hematocrit of 45% was filled into the gap and the shear rate was varied from 0 to 100 s^−1^. Nondimensionalized by an average RBC diameter (*D* = 8 *μ*m), the computational domain was modelled as a small box inside the experimental domain [7]; see Figure 8 (in the experiment, the top plate is fixed and the bottom plate is moving [7]). This box size was deemed large enough as long as the largest structure (aggregation network) is not suppressed by the utilisation of periodic boundary conditions. The computational domain was divided into 96 blocks in space for parallel computation.

For the fluid, the bottom plate was set as a stationary nonslip wall, and the top plate was set to a nonslip wall moving at a constant velocity. Periodic boundaries were adopted in both streamwise and spanwise directions. The fluid media were considered to be blood plasma with physical properties set as normal values; see Table 2. The shear rate *γ* was calculated by *γ* = Us/*h*, where Us is the shearing velocity of the top plate and *h* is the thickness between the top and the bottom plate. The Reynolds number was calculated based on the shearing velocity Us and the flow thickness *h*. The total number of RBCs was 49,512, which yielded a hematocrit of around 45%.

**Figure 9:**
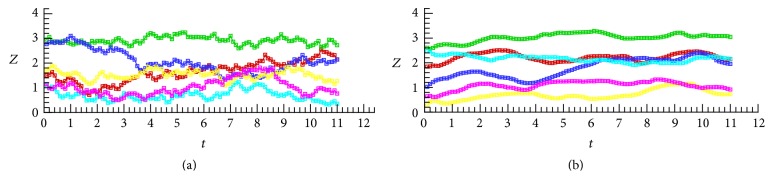
Trajectory of 6 sampled RBCs expressed in* Z* coordinate: (a) high shear rate (*γ* = 100/s) and (b) low shear rate (*γ* = 10/s); high shear rates tend to show more high frequency fluctuations, while low shear rates show smooth variations.

During the simulation, 6 RBCs were randomly sampled and the coordinates of their centers were recorded; see Figure 9. Both the high and low shear rate results show time varying fluctuations, and the most significant difference between them is the fluctuation frequency. The high shear rate results show high frequency irregular fluctuations much more than those of the low shear ones. The low shear rate results show a smooth time varying tendency with large time periods. This can be explained as the appearance of aggregates, where the individual RBCs moving, attached to aggregation blocks, have much larger sizes. However, the motion of a block of aggregated RBCs carried by blood flow has not been previously investigated, and such studies are necessary because in many cases human RBCs flow as aggregates, rather than individual cells.

**Figure 10:**
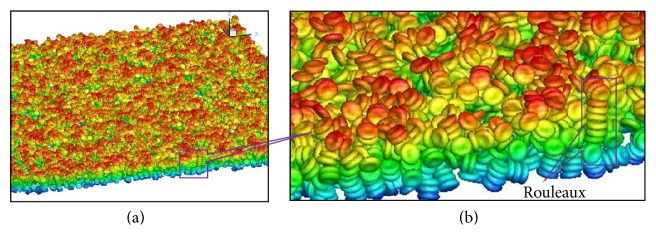
Three-dimensional view of the simulation results at shear rate 60 s^−1^: (a) overall view and (b) local view of the rouleaux structures.

The numerical simulation provided full information of the position, movement, deformation, and aggregation of the RBCs in the shear flow. See Figure 10 for the simulated results of RBCs under a shear rate of 60 s^−1^. It can be seen that RBCs aggregate face to face to form coin-stack-like structures, which are called rouleaux. The formation of rouleaux can be explained by the unique discoid shape of the cells. The massive data produced by numerical simulations such as in the present study can be used for statistical analysis and for further investigation of the effect of aggregation in healthy and diseased blood flow.

**Figure 11:**
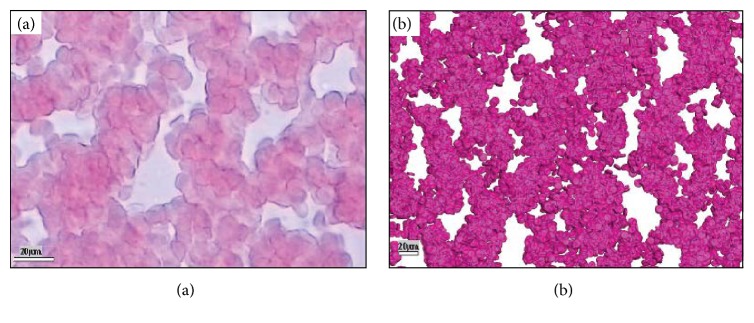
Comparison between experimental and simulation results. (a) Experiment snapshot at shear rate 10/s [7]. (b) Simulation results at shear rate 10/s.

The large scale of the simulation has enabled a macroscale verification and investigation into the overall characteristics of RBC aggregations to be carried out rather than just focusing on several individual cells. The comparisons show apparent similarities of blood microstructure between the experiment and the simulation; see Figure 11 [14]. Under the shear rate of 10 s^−1^, both the experiment and the simulation show significant aggregation of RBCs. In this case, the adhesion forces play dominant roles compared with the shearing force by the fluid which tends to separate the cells. The simulated results agree well with the experiment as shown by Figures 11(a) and 11(b). For human blood with a hematocrit of 45%, aggregation characteristics such as those simulated in the present study have been widely reported in clinical experiments [1, 2]. However, under a shear rate as high as 100/s, most of the aggregation structures will be destroyed and the RBCs distribute more uniformly in space—this was also verified by both experiments and simulations [12].

**Figure 12:**
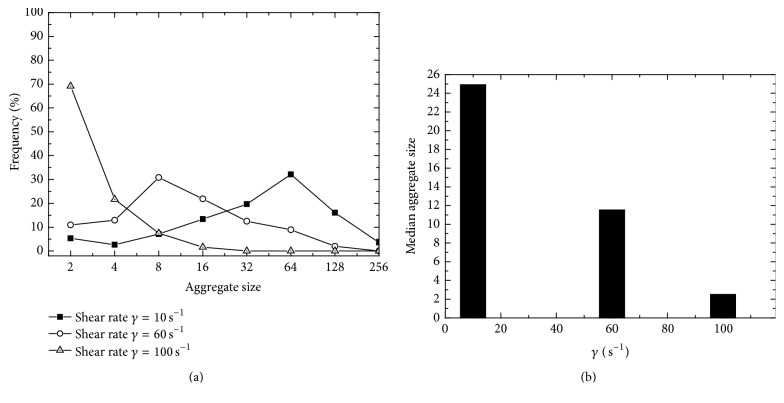
Aggregate sizes of RBCs based on simulation results. (a) Aggregate size distributions at equilibrium states. (b) Median aggregate size under 3 different shear rates.

For quantitative analysis of the RBC aggregations, the aggregate degrees were estimated by the aggregate size, namely, the average number of RBCs per aggregate [29]. The distribution of number of RBCs in each aggregate (aggregate size) was examined by categorizing aggregates into ranges of aggregate sizes on a logarithmic scale and categories were labeled by the truncated smallest size of the range; see Figure 12(a). There is a clear tendency that, with increasing flow shear rate, the peak values of aggregate size frequencies offset to the left. For shear rate of 10 s^−1^, around 33% aggregates have a size ranging from 64 to 128; however, for a high shear rate of 100 s^−1^, around 70% of aggregates have a size ranging from 2 to 4. The median aggregate size drops dramatically with the shear rate; see Figure 12(b). Such tendencies were also observed in [29], although the data cannot be quantitatively compared due to differences in shear rates and fluid viscosity.

## 4. Conclusions

A three-dimensional computational model has been developed to numerically simulate the deformation and aggregation of RBCs by coupling the interaction between the fluid and the deformable solid membrane of the RBC using continuum mechanics. The capability of the proposed model was verified against experimental data and the major contribution of the work reported in this paper is the establishment of a computational framework that can fully resolve the deformation and aggregation of individual RBCs up to a number of 49,152. This was achieved with full parallelization of the fluid-solid coupled solver using spatial decomposition and high performance computers. The large-scale feature of the simulations has enabled a macroscale verification and investigation into the overall characteristics of RBC aggregations to be carried out. The results from the simulations have been compared with experimental data with very good agreement. Both the experiment and the simulation show a uniform distribution of RBCs under high shear rates (*γ* = 100/s, *γ* = 60/s) and large aggregation structures under a low shear rate (*γ* = 10/s). The median aggregate size drops dramatically with the shear rate. Simulations of a Poiseuille flow formed in a tube with a confined throat show that, in stenosed vessels or micro peristaltic pumps, the hematocrit may not only vary both in the longitudinal direction but also fluctuate with time. Further investigations should be carried out to interpret this phenomenon from the aspect of mechanics and energy.

## Figures and Tables

**Table 1:** CPU usage for different parts of the coupled solver.

Fluid solver	RBC deformation	RBC contact detection	RBC interactions	Data exchange
20%	8.8%	2.2%	60%	9%

**Table 2:** Physical properties of the fluid (plasma) and solid (RBC).

Property	Symbol	Value
Fluid density	*ρ*_*f*_	1025 kg/m^3^
Fluid viscosity	*ν*	1.46 × 10^−6^ m^2^/s
Shear rate	*γ*	10 s^−1^, 60 s^−1^, 100 s^−1^
Reynolds number	Re	6.16 × 10^−4^–6.16 × 10^−2^
Gravity	*g*	9.81 m/s^2^
Hematocrit	Ht	45%
RBC density	*ρ*_*s*_	1125 kg/m^3^
RBC diameter	*D*	8.0 *µ*m
RBC thickness	*h*	2.24 *µ*m
Membrane modulus	*C*1	2.57 × 10^6^ dyn/cm^2^
Membrane modulus	*C*2	2.57 × 10^5^ dyn/cm^2^
Work of adhesion	*σ*	8 *µ*J
